# Lipase-Catalyzed Baeyer-Villiger Oxidation of Cellulose-Derived Levoglucosenone into (*S*)-γ-Hydroxymethyl-α,β-Butenolide: Optimization by Response Surface Methodology

**DOI:** 10.3389/fchem.2016.00016

**Published:** 2016-04-19

**Authors:** Andreia R. S. Teixeira, Amandine L. Flourat, Aurelien A. M. Peru, Fanny Brunissen, Florent Allais

**Affiliations:** ^1^Chaire Agro-Biotechnologies Industrielles, AgroParisTechReims, France; ^2^UMR GENIAL, AgroParisTech, Institut National de la Recherche Agronomique, Université Paris-SaclayMassy, France; ^3^UMR 1318 IJPB, AgroParisTech, Institut National de la Recherche Agronomique, Université Paris-SaclayVersailles, France; ^4^UMR 782 GMPA, AgroParisTech, Institut National de la Recherche Agronomique, Université Paris-SaclayThiverval-Grignon, France

**Keywords:** response surface methodology, reaction optimization, Bayer-Villiger bio-oxidation, lipase, enzymatic reaction, levoglucosenone

## Abstract

Cellulose-derived levoglucosenone (**LGO**) has been efficiently converted into pure (*S*)-γ-hydroxymethyl-α,β-butenolide (**HBO**), a chemical platform suited for the synthesis of drugs, flavors and antiviral agents. This process involves two-steps: a lipase-catalyzed Baeyer-Villiger oxidation of **LGO** followed by an acid hydrolysis of the reaction mixture to provide pure **HBO**. Response surface methodology (RSM), based on central composite face-centered (CCF) design, was employed to evaluate the factors effecting the enzyme-catalyzed reaction: pka of solid buffer (7.2–9.6), **LGO** concentration (0.5–1 M) and enzyme loading (55–285 PLU.mmol^-1^). Enzyme loading and pka of solid buffer were found to be important factors to the reaction efficiency (as measured by the conversion of **LGO**) while only the later had significant effects on the enzyme recyclability (as measured by the enzyme residual activity). **LGO** concentration influences both responses by its interaction with the enzyme loading and pka of solid buffer. The optimal conditions which allow to convert at least 80% of **LGO** in 2 h at 40°C and reuse the enzyme for a subsequent cycle were found to be: solid buffer pka = 7.5, **[LGO]** = 0.50 M and 113 PLU.mmol^-1^ for the lipase. A good agreement between experimental and predicted values was obtained and the model validity confirmed (*p* < 0.05). Alternative optimal conditions were explored using *Monte Carlo* simulations for risk analysis, being estimated the experimental region where the **LGO** conversion higher than 80% is fulfilled at a specific risk of failure.

## 1. Introduction

Driving forces for the global trend of using clean renewable sources in the production of valuable chemical are the inevitable decline of fossil fuels and a more demanding legislation regarding the disposal of industrial wastes. In this context, lignocellulosic biomass is envisaged as an interesting source to produce highly valuable synthons due to its low cost and high availability. Thermochemical processing, phosphoric acid-catalyzed pyrolysis in particular, is the simplest way to efficiently convert lignocellulosic biomass into its degradation products (Huber et al., 2006; Babu, 2008). However, the use of strong acids is one of the drawbacks of such process, mainly associated to its inefficient recovery and environmental concerns related to water and air pollution (Wei et al., 2014). Furacell Process developed by Circa is claimed to be an easily scalable catalytic thermochemical process with no harmful effluents which converts renewable cellulose into a very simple mixture of easily separable products, comprising levoglucosenone (**LGO**), char and water reusable in the process (Court et al., 2012).

**LGO** is a multifunctional C6-monomer suited for organic synthesis (Miftakhov et al., 1994; Sarotti et al., 2012). Indeed, **LGO** is a chiral synthon that can be used in the synthesis of a wide variety of biologically active compounds such as pharmaceutical ingredients, agrochemicals, polymers, or specialty chemicals (Miftakhov et al., 1994; Budarin et al., 2011). Among them, (*S*)-γ-hydroxymethyl-α,β-butenolide (aka **HBO**) is certainly the most interesting since it is a precursor of many drugs (Enders et al., 2002), flavors (Kawakami et al., 1990), and antiviral agents (Flores et al., 2011).

**Figure 1:**
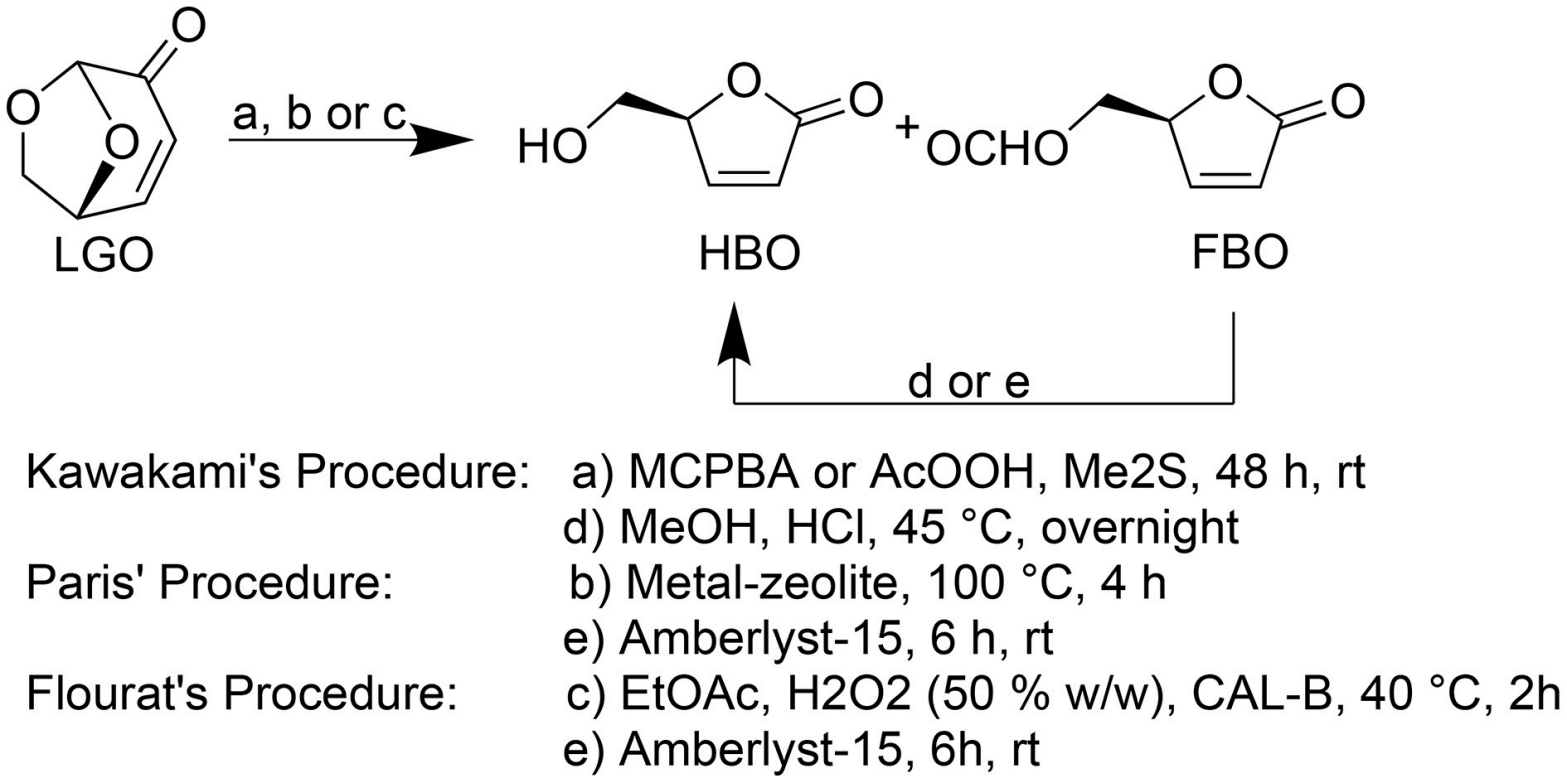
**Baeyer-Villiger oxidation of LGO into HBO and FBO following Kawakami (Kawakami et al., 1990), Paris (Paris et al., 2013), and Flourat's (Flourat et al., 2014) procedures**.

Kawakami (Kawakami et al., 1990) and Paris (Paris et al., 2013) reported the most efficient methods for **HBO** synthesis, being obtained high overall yields (ca. 80–90%, Figure 1).

In both methods, **HBO** is produced through a Baeyer-Villiger oxidation of **LGO** followed by an acid hydrolysis to convert formate lactone (**FBO**), a reaction by-product, into **HBO**. Kawakami*et al*. (Kawakami et al., 1990) used peracids (such as peracetic acid or *m*-chloroperbenzoic acid) and Me_2_S during 48 h followed by an HCl-mediated hydrolysis to provide pure **HBO**. Paris et al. (2013) developed a method to produce **HBO** in only 4 h using metal-based zeolites as catalysts to oxidize **LGO** and Amberlyst-15 as acid resins to promote the subsequent acid hydrolysis. However, the complexity and high cost inherent to zeolites synthesis may compromise the technical and economic viability of such process (Perot and Guisnet, 1990).

**Figure 2:**
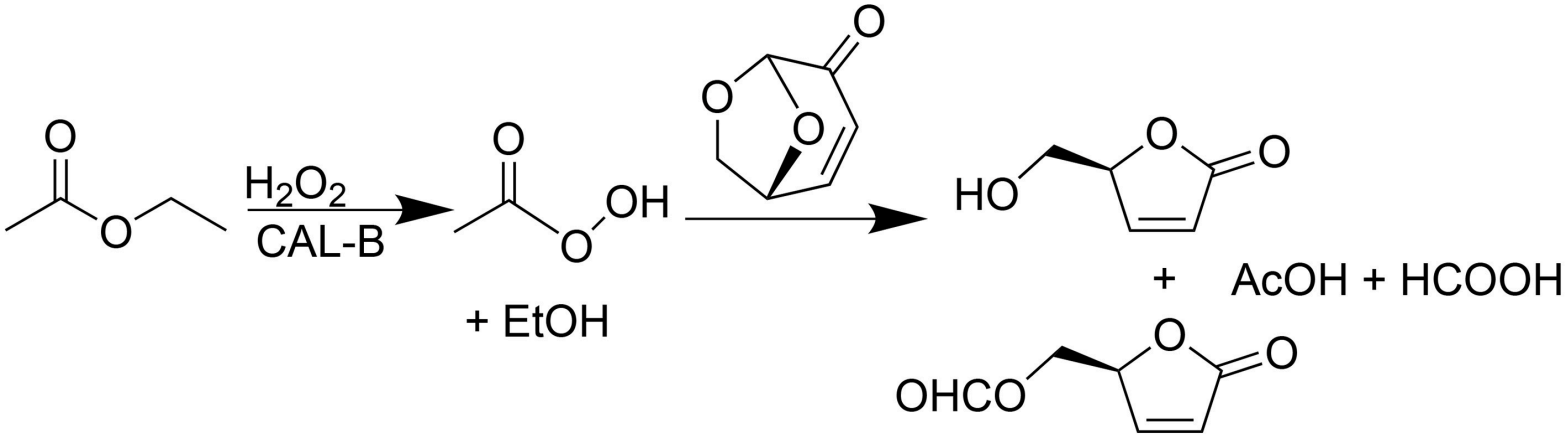
**Lipase-mediated Baeyer-Villeger oxidation of LGO into HBO and FBO using AcOEt as an acyl donor and H_**2**_O_**2**_ as an oxidant**.

The use of lipases as biocatalyst seemed to be a promising greener alternative, providing, in addition, a cost-efficient transformation. In a recent publication (Flourat et al., 2014), we reported an efficient chemo-enzymatic process for the production of **HBO** with high yields (> 80%). The first step involved a Baeyer-Villiger oxidation of **LGO** mediated by a commercial immobilized lipase from *Candida antarctica* (CAL-B, Novozyme® 435), under the presence of a solid buffer and using ethyl acetate and hydrogen peroxide as acyl donor and oxidant, respectively (Figure 2). After 2 h of reaction, the resulting mixture of **FBO** and **HBO** was hydrolyzed under acid conditions, using Amberlyst-15, to provide pure **HBO**.

Response surface methodology (RSM) is an effective tool for optimizing a range of processes (Montgomery, 2008) and evaluate the interactions of multiple parameters, being established in addition a prediction model based on statistics (Montgomery, 2008). RSM based on central composite face-centered (CCF) design was employed to found the conditions which allow to maximize simultaneously the conversion of **LGO** and the enzyme residual activity. The present work focuses on the study of reaction parameters (solid buffer pka, **LGO** concentration and enzyme loading) that may affect the conversion of **LGO** into **HBO** and the enzyme recyclability (measured by the enzyme residual activity) in order to establish their relationships and, if possible, further optimize the conversion obtained in an earlier authors' publication (Flourat et al., 2014); 83%. The temperature was set at 40°C to minimize the **LGO** degradation (reactant specie due to its acetal moiety) and to avoid the risk of explosion linked to the *in-situ* formation of peracetic acid. The RSM model allowed explore alternative optimal conditions and using *Monte Carlo* simulations the risk of failure could be determined.

## 2. Experimental

### 2.1. Reagents

Cellulose-derived levoglucosenone (**LGO**) was kindly provided by CIRCA Group (Knoxfield Victoria, Australia). Novozyme® 435 (CAL-B, lot no. SLBF9301 V, 9120 PLU g^-1^), hydrogen peroxide (50% w/w), biological solid buffers (MOPS, TAPS, CAPSO, HEPES and their sodium salt), lauric acid (99%) and 1-propanol were purchased from Sigma-Aldrich. Ethyl acetate (analytical grade), hexane (ACS reagent) and acetonitrile (HPLC grade) were purchased from Thermofisher Scientific. Ultra-pure laboratory grade water (MilliQ, 18.2 megaOhms, 25°C) was employed for HPLC analysis.

### 2.2. Baeyer-Villiger oxidation of (-)-levoglucosenone (LGO)

Lipase-mediated Baeyer-Villiger oxidation of **LGO** is schematically represented in Figure 2. Reactions were carried out in sealed erlenmeyers (50 mL) to avoid solvent evaporation. **LGO** (250 mg, 1 equiv.) was dissolved in different volumes of ethyl acetate (C = 0.5–1 M). Biological solid buffers along with their sodium form were added (20 mg mL^−1^ each) in order to set the pka (7.5 < pka < 9.6) and as consequence control the protonation state of the enzyme. It should be noted that unlike the organic soluble buffers, each buffer pair will set a fixed value of the relevant ionization parameter (Partridge et al., 2001). Different amounts of lipase (CAL-B) were added accordingly to those indicated in **Table 2**, followed by the addition of hydrogen peroxide (1.2 equiv., 50% w/w) at once. The mixture was incubated at 40°C and stirred using an orbital shaker (ThermoScientific, MaxiQ400) at 250 rpm for 2 h. Samples (10 μ*L*) were collected and diluted with 1.5 mL of acetonitrile for **LGO** quantification by HPLC analysis. The conversion of **LGO** was defined as the molar ratio of the remaining **LGO** and the **LGO** added in the beginning.

### 2.3. Determination of enzyme residual activity

After reaction, the enzyme was recovered by filtration, washed with ethyl acetate (10 mL) and hexane (10 mL), dried in an oven at 40°C for 1 h, and then kept in dessicator overnight and under vacuum. Three enzyme samples (ca. 14 mg each) were collected, weighed in a 20 mL vial and kept at 60°C. A solvent free equimolar solution of lauric acid and 1-propanol with 3% w/w of water was prepared and incubated at 60°C. After 1 h, 5 g of the above solution were added to the enzyme and stirred in an incubating orbital minishaker (VWR) at 400 rpm and 60°C for 15 min. Samples (2 μ*L*) were collected, weighed in a GC-vial and diluted with 1 mL of hexane. Lauric acid conversion was determined by GC-MS. Unit definition: 1 PLU = 1 μ*mol* of 1-propyl laurate formed per gram of enzyme per minute at 60°C.

### 2.4. GC-MS method

Lauric acid (used to determine the enzyme residual activity) was quantified by a GC-MS system which consisted of an Agilent GC 5975 coupled with MS 7890 in electron impact mode with electron energy set at 70 eV and a mass range at *m/z* (30–350 amu). A HP5-MS capillary column (Agilent, 30 m × 0.25 mm, 0.25 μ*m*) was used for chromatographic separation. Injection was performed at 280°C in split mode (40:1), being injected 1 μ*L* of each sample. The oven temperature program was the following: from 60°C held for 1 min, then rinse until 325°C at 20°C/min with a 5 min hold. Hydrogen flow rate was set at 1.2 mL/min. The mass detector was set as follows: source and quad temperatures at 230 and 150°C, respectively. A calibration curve was performed each time, with pure lauric acid (0.2–4.5 mg/mL) in hexane. Typical retention time for lauric acid and 1-propyl laurate were 7.22 min and 7.92 min, respectively.

### 2.5. HPLC method

(-)-Levoglucosenone (**LGO**) was quantified by HPLC (Thermofisher Ultimate 3000) on a Syncronis aQ column (250 × 4.6 mm, 5 μ*m*, Thermoscientific) with Milli-Q water (solvent A) and acetonitrile (solvent B) as mobile phase. The flow rate and temperature were set at 0.8 mL/min and 30°C, respectively. The gradient elution was as follows: isocratic at 85% A (0–5 min), from 85 to 90% A (5–10 min), isocratic at 90% A (10–15 min), from 90% A to 85% A (15–20 min). Adequate detection was obtained with a diode array detector (DAD) set at 220 nm. Injection volume in HPLC injector was set at 10 μ*L*. Samples for HPLC analysis were prepared by diluting 10 μ*L* of the reaction mixture in 1.5 mL of acetonitrile. The peaks of **LGO** were identified and quantified using a standard curve prepared in acetonitrile. Typical retention time for **HBO**, **FBO** and **LGO** were 3.73, 3.87, and 8.40 min, respectively.

### 2.6. Experimental design and statistical analysis

RSM, based on a 3-factor-3-level CCF design, was employed to determine the parameters affecting the Baeyer-Villiger oxidation of **LGO** as well as to found the optimal set of conditions. CCF is a good choice from a practical point of view, even inducing some correlations between the quadratic terms. These correlations will induce a slight increase in the confidence intervals but the model will still be able to estimate the quadratic effects.

A short reaction time of 2 h was set in order to minimize the residence time of the enzyme and limit the contact with the inhibitors as well as to minize the residence time of **LGO** inside the reactor and, thus, minimizing **LGO** degradation. The temperature was set at 40°C for the same reason but also to minimize the risk of explosion linked to the presence of peracetic acid (intermediary specie).

Table 1 presents the independent variables (*x*_*i*_), levels and experimental design in terms of uncoded and coded (transformation of each studied real value into coordinates inside a scale with dimensionless values). The variation ranges of each variable were fixed taking into account specific constraints:

- Following the manufacture (Novozymes, 2015) the optimal pka for the lipase CAL B lies between 5 and 9. However, the pka buffer cannot be acid (<7) in order to avoid the degradation of **LGO** (acetal moieties reactive under such conditions). Therefore, the effect of pka in the enzyme protonation state was assessed with MOPS (pka = 7.2) and CAPSO (pka = 9.6), using TAPS (pka = 8.4) as a central point.
- A lower solvent volume to obtain higher **LGO** concentrations implies the presence of inhibitors (peracetic acid, acetic acid and formic acid) at higher concentrations. Moreover, the use of high **LGO** concentrations (> 1 M) leads to a decrease in the **LGO** conversion and (mainly) in the enzyme activity (Flourat et al., 2014). Instead, a low concentration of substrates implies the use of large quantities of solvents, which may compromise the economic sustainability of a process. Thus, **LGO** concentration was varied between 0.5 and 1 M.
- Enzymes must be used at catalytic quantities to assure the economic sustainability of the process. Thus, the enzyme loading was varied between 55 and 285 PLU.mmol^−1^, which corresponds to 2 and 10 % (w/w), respectively.

**Table 1:** **Independent variables and levels used for CCF design**.

**Variables**	**Level**		
	**−1**	**0**	**1**
Solid buffer pka	MOPS (7.2)	TAPS (8.4)	CAPSO (9.6)
Enzyme loading (PLU.mmol^-1^)	55	170	285
**LGO** concentration (M)	0.5	0.75	1

Table 2 presents the runs set by a CCF design and the respective experimental responses obtained for **LGO** conversion (*Y*_1_) and enzyme residual activity (*Y*_2_). CCF design consists of 8 factorial points, 6 axial points (two axial points on the axis of each design variable at a distance 1 from the design point) and 3 central points (Eriksson et al., 2008), making a total of 17 runs. Duplicates in each point were performed in order to obtain a more precise model (total of 34 runs). To avoid bias, all runs were performed in a totally random order. Additional runs (intermediary points) were performed to validate the model and later added to the experimental data for model refining.

**Table 2:** **Central composite face-centered (CCF) design and experimental responses**.

**Run/Replicate ^a^**	**Solid buffer (pka)**	**Enzyme loading (PLU.mmol^−1^ of LGO)**	**[LGO] (M)**	**LGO conversion (%)**	**Enzyme residual activity (%)**
	***x*_1_**	***x*_2_**	***x*_3_**	***Y*_1_**	***Y*_2_**
1/18	MOPS (7.2)	55	0.5	57.4/68.0	88.5/82.0
2/19	CAPSO (9.6)	55	0.5	79.9/79.7	63.6^b^/37.6
3/20	MOPS (7.2)	285	0.5	81.3/87.0	85.3/81.0
4/21	CAPSO (9.6)	285	0.5	87.5/90.8	52.7/57.3
5/22	MOPS (7.2)	55	1.0	74.8/74.5	70.3/76.9
6/23	CAPSO (9.6)	55	1.0	47.3/63.9	60.2/60.7
7/24	MOPS (7.2)	285	1.0	81.5/91.6	51.2/44.8
8/25	CAPSO (9.6)	285	1.0	87.2/90.4	69.2/52.4^b^
9/26	MOPS (7.2)	170	0.75	84.4/87.2	72.4/73.4
10/27	CAPSO (9.6)	170	0.75	90.0/88.9	68.9/67.0
11/28	TAPS (8.4)	55	0.75	73.9/82.0	73.1/63.1
12/29	TAPS (8.4)	285	0.75	67.8^b^/91.3	74.8/50.6
13/30	TAPS (8.4)	170	0.5	86.6/87.6	61.8/61.1
14/31	TAPS (8.4)	170	1.0	88.4/89.5	12.6^b^/67.5
15/32	TAPS (8.4)	170	0.75	87.9/90.2	65.7/68.5
16/33	TAPS (8.4)	170	0.75	93.8/90.3	74.9/74.1
17/34	TAPS (8.4)	170	0.75	92.4/89.9	76.8/75.8
**ADDITIONAL RUNS**					
35/36	MOPS (7.2)	152	0.70	78.0/80.8	78.2/79.4
37/38	CAPSO (9.6)	80	0.94	72.5/73.6	70.0/71.6
39	HEPES (7.5)	120	0.65	79.7	76.5

a *Runs performed in a totally random order*.

b *Outlier: observation point that is distant from other observations. Excluded from the data set*.

In order to find a suitable approximation for the true functional relationship between independent variables and the response surface, a second-order polynomial Equation (1) was used, being expressed as:

(1) $$\begin{array}{l} Y=\beta_{0}+\overset{3}{\underset{i=1}{\sum }}\beta_{ki}x_{i}+\overset{3}{\underset{i=1}{\sum }}\beta_{kii}x_{i}^{2}+\overset{2}{\underset{i=1}{\sum }}\overset{3}{\underset{j=i+1}{\sum }}\beta_{kij}x_{i}x_{j} \end{array}$$

where *Y*_*i*_ represents the response *i* (**LGO** conversion and enzyme residual activity in this study), *x*_*i*_ are the coded independent variables, β_0_ is a constant coefficient, and β_*ki*_, β_*kii*_, and β_*kij*_ are the linear, quadratic, and interaction coefficients, respectively.

The variable levels *X*_*i*_ were scaled and centered (coded) according to the equation below such that *X*_0_ corresponded to the central value:

(2) $$\begin{array}{l} x_{i}=\frac{X_{i}-X_{0}}{\Delta X_{i}}\;i=1,2,3,\ldots ,k \end{array}$$

where *x*_*i*_ is the dimensionless value of an independent variable, *X*_*i*_ is the real value of an independent variable, *X*_0_ is the real value of an independent variable at the center point, and Δ*X*_*i*_ is the step change.

Modde v.10.1 sofware (Umetrics AB, Sweden) was used to generate the CCF design and analyze experimental data by RSM. Regression coefficients were determined by multiple linear regression (MLR). The significant parameters in the model were found by analysis of their *p*-value. The model validation was based on the variance (ANOVA) for each response, namely, by the analysis of *R*^2^, *Q*^2^, and *lack of fit* (*LOF*) test. *R*^2^ measures how well the regression model fits the experimental data, *Q*^2^ shows an estimate of the future prediction precision, and, *LOF* assess whether the models error is comparable to the replicate error.

## 3. Results and discussion

### 3.1. Preliminary study

In our previous study (Flourat et al., 2014) we reported the ability of commercial immobilized *Candida antarctica* lipase (Novozyme® 435) to oxidize **LGO** (Figure 2). High conversions of **LGO** (> 83%) were obtained after 2 h, using a low enzyme loading (113 PLU.mmol^−1^) and a **LGO** concentration of 0.75 M. Such mild conditions allowed to reuse the enzyme for a second reaction cycle without a significant loss of enzyme activity.

The optimization was conducted using the One Variable At a Time (OVAT) method, being optimized the temperature, **LGO** concentration, enzyme loading, nature of buffer (solid/liquid and pka) as well as the nature and quantity of the oxidizing agent.

Results showed that at low temperatures (<40°C) the final **LGO** conversion is compromised, being, although, similar at 40 and 60°C (after 8 h of reaction). However, there are other reasons to remain at 40°C than an obvious energy saving: the **LGO** degradation (due to its acetal moiety) as well as the risk of explosion linked to the presence of peracetic acid (intermediary specie). For all this reasons, the temperature was set at 40°C in this work even having to ignore interactions with other variables.

Moreover, it was showed that a low-water media (resulted in using solid buffers) leaded to higher conversion of **LGO** probably due to a higher stability of the enzyme and inhibition of secondary reactions. Unfortunately in this reaction, the water activity cannot be actively controlled due to high water content of commercial hydrogen peroxide solution (50% w/w).

As summary, the previous study (Flourat et al., 2014) suggested that only three variables had significant effect on the **LGO** conversion. These variables were the buffer pka (*x*_1_), the enzyme loading (*x*_2_) and the **LGO** concentration (*x*_3_). The level values of variables were chosen in such a way that their limits were as wide as possible and that contains the optimal values determined using the OVAT method (Table 1). The levels of the three other variables with a small effect on the oxidation reaction were fixed as follow: temperature: 40°C, reaction time: 2 h, nature and quantity of the oxidizing agent: hydrogen peroxide (H_2_O_2_) at 1.2 equiv. (relative to **LGO**).

### 3.2. Assessing the design orthogonality

The condition number is a parameter that assesses the sphericity of the design, thus, the orthogonality. Formally, the condition number is the ratio of the largest and the smallest singular values of the X-matrix, that is, the matrix of the factors extended with higher order terms. As a thumb rule for an optimization (Eriksson et al., 2008), the condition number should be lower than 8. The condition number obtained in our model was 4.1 for **LGO** conversion and 1.6 for the enzyme activity. Such low values demonstrate well the orthogonality of the selected design, thus, the adequacy of the design selected.

As can be seen in Table 2, additional runs were performed. Such procedure allowed improving the model quality at an exploratory level, however, without compromising the model orthogonality.

### 3.3. Optimization by design of experiments (DOE)

The analysis of experimental data through DOE consists of four primary stages. The first stage, *evaluation of raw data*, focuses on identifying regularities and peculiarities in the experimental data. The second stage, *regression analysis*, involves the calculation of the model linking the variables and response(s) together, and is followed by a third stage, the *model interpretation*. Finally, in the fourth stage, *use of regression model*, the model obtained is used to predict the optimal experimental conditions to maximize/minimize the response(s).

#### 3.3.1. Testing the normality for raw data evaluation

In regression analysis, it is advantageous if data of a response are normally distributed. This improves the efficiency of data analysis, and enhances model validity and inferential reliability. It is not recommended to apply regression analysis to a response with heavy tails as originally observed for **LGO** conversion (*Y*_1_) and enzyme residual activity (*Y*_2_) responses (Figure S1), since that would correspond to assigning the extreme measurement an undue influence in the modeling (Eriksson et al., 2008). Normality can be assessed to some extent by obtaining skewness and kurtosis values. Skewness is a measure of the asymmetry of the probability distribution of a random variable about its mean. Kurtosis is a measure of the “peakedness” of the probability distribution of a random variable. In other words, kurtosis indicates how tall and sharp the central peak is, relative to that of a standard bell curve. As a general rule of thumb the skewness and kutosis values should range between −0.5 and 0.5 in a normal distribution (Montgomery, 2008). If these values are not included within such range, a transformation of the response should be performed (see Supplementary Material for more details). Table 3 compares these values for both responses before and after negative logarithmic transformation.

**Table 3:** **Skewness and kurtosis values for LGO conversion (***Y***_**1**_) and enzyme residual activity (***Y***_**2**_) responses before and after a negative logarithmic transformation**.

**Test**	**Before**		**After**	
	***Y*_1_**	***Y*_2_**	***Y*_1_**	***Y*_2_**
Skewness	−1.2	−0.67	−0.46	0.32
Kurtosis	1.1	0.32	−0.43	0.50

As shown in Table 3, skewness and kutosis values are within the range after response transformation. In this transformation, each measured value is subtracted from the maximum value (100, for variables expressed in percentages), and then the negative logarithm is applied (see Supplementary Material for more details, including other tools for measure the raw data normality).

#### 3.3.2. Regression analysis

The next stage consists of fitting the second-order polynomial Equation (**1**) to the experimental data (Table 2) and determining the significant coefficients for each response.

Designs with a low condition number mean having low correlations among the terms in the model. As can be seen in Table 4, significant correlations are only observed between variables and responses (values highlighted in bold). An exception for the correlations between quadratic terms, as a direct consequence of the design selection (CCF). However, it should be highlighted that these correlations induce a slight increase in the confidence intervals but the model will still be able to estimate the quadratic effects.

**Table 4:** **Correlation matrix**.

	***x*_1_**	***x*_2_**	***x*_3_**	*x*_1*_x_1_	*x*_2*_x_2_	*x*_3*_x_3_	*x*_1*_x_2_	*x*_1*_x_3_	*x*_2*_x_3_	***Y*_1_**	***Y*_2_**
*x*_1_	1	−0.048	0.104	0.004	0.097	0.100	−0.086	0.048	−0.072	0.088	**−0.505**
*x*_2_	−0.048	1	−0.070	0.010	0.040	0.015	0.051	−0.067	0.050	**0.674**	−0.182
*x*_3_	0.104	−0.070	1	0.010	0.026	0.040	−0.072	0.051	−0.075	−0.052	−0.210
*x*_1_^*^x_1_	0.004	0.010	0.010	1	**0.492**	**0.382**	−0.041	0.060	−0.043	**−0.463**	0.078
*x*_2_^*^x_2_	0.097	0.040	0.026	**0.492**	1	**0.578**	−0.037	−0.035	−0.035	**−0.423**	−0.179
*x*_3_^*^x_3_	0.100	0.015	0.040	**0.382**	**0.578**	1	0.011	−0.011	−0.103	**−0.361**	−0.120
*x*_1_^*^x_2_	−0.086	0.051	−0.072	−0.041	−0.037	0.011	1	−0.084	0.047	0.123	0.274
*x*_1_^*^x_3_	0.048	−0.067	0.051	0.060	−0.035	−0.011	−0.084	1	−0.020	**−0.303**	**0.544**
*x*_2_^*^x_3_	−0.072	0.050	−0.075	−0.043	−0.035	−0.103	0.047	−0.020	1	0.149	−0.067
*Y*_1_	0.088	**0.674**	−0.052	**−0.463**	**−0.423**	**−0.361**	0.123	**−0.303**	0.149	1	−0.240
*Y*_2_	**−0.505**	−0.182	−0.210	0.078	−0.179	−0.120	0.274	**0.544**	−0.067	−0.240	1

*Significant correlations are identified in bold*.

The standard approach for selecting significant coefficients for each response is based on their *p*-value (Eriksson et al., 2008; Montgomery, 2008): a *p*-value lower than 0.05 means that the coefficient is significant (Tables 5, 6, values highlighted in bold). The coefficients centered and scaled (Coeff. SC, referring to the coded −1 to +1 unit) as well as their standard error (Std. Err.), *p*-value and confidence interval at 95% (CI) are listed in the Tables 5, 6 for the **LGO** conversion and the enzyme residual activity models, respectively.

**Table 5:** **Model coefficients [centered and scaled (SC)], their standard error (Std. Err.), ***p***-value and confidence interval at 95% (CI) for the LGO conversion model**.

**Yield**	**Coeff. SC**	**Std. Err**.	***p*-value**	**CI _95*%*_ (±)**
Constant	0.992	0.029	**3.62** × 10^−24^	0.061
*x*_1_	0.050	0.020	**0.020**	0.041
*x*_2_	0.201	0.022	**6.08** × 10^−10^	0.045
*x*_3_	0.004	0.021	0.841	0.043
*x*_1_^*^x_1_	−0.125	0.039	**3.59** × 10^−3^	0.081
*x*_2_^*^x_2_	−0.119	0.044	**0.011**	0.089
*x*_3_^*^x_3_	−0.057	0.039	0.165	0.081
*x*_1_^*^x_2_	0.020	0.024	0.417	0.049
*x*_1_^*^x_3_	−0.082	0.024	**1.74** × 10^−3^	0.048
*x*_2_^*^x_3_	0.028	0.024	0.245	0.049

*Significant p-values (< 0.05) are identified in bold*.

**Table 6:** **Model coefficients [centered and scaled (SC)], their standard error (Std. Err.), ***p***-value and confidence interval at 95% (CI) for the enzyme residual activity model**.

**Yield**	**Coeff. SC**	**Std. Err**.	***p*-value**	**CI _95*%*_(±)**
Constant	−1.48	0.029	**2.49** × 10^−28^	0.060
*x*_1_	−0.090	0.020	**1.73** × 10^−4^	0.043
*x*_2_	−0.032	0.022	0.158	0.046
*x*_3_	−0.039	0.022	0.090	0.046
*x*_1_^*^x_1_	0.035	0.039	0.377	0.080
*x*_2_^*^x_2_	−0.011	0.043	0.790	0.088
*x*_3_^*^x_3_	−0.040	0.042	0.343	0.086
*x*_1_^*^x_2_	0.059	0.025	**0.025**	0.051
*x*_1_^*^x_3_	0.123	0.024	**2.67** × 10^−5^	0.050
*x*_2_^*^x_3_	−0.027	0.025	0.284	0.051

*Significant p-values (< 0.05) are identified in bold*.

For the model of **LGO**, following the *p*-value analysis of each coefficient, **LGO** concentration (*x*_3_), the respective quadratic term (*x*_3_ * *x*_3_), the interaction between pka, the enzyme loading (*x*_1_ * *x*_2_) as well as the interaction between enzyme loading and **LGO** concentration (*x*_2_ * *x*_3_) are not significant terms. For the model of enzyme residual activity, the enzyme loading (*x*_2_), the **LGO** concentration (*x*_3_), all quadratic terms as well as the interaction between enzyme loading and **LGO** concentration (*x*_2_ * *x*_3_) are not significant terms. As result, the fitted model for each response, expressed in coded variables (scaled and centered), may be represented by the following equations (see in Supplementary Material the model equations in uncoded units, as well as their 3D graphical representation, Figure S6):

(3) $$\begin{array}{r} -log_{10}\left(100-Y_{1}\right)=-0.993+0.052x_{1}+0.208x_{2}-0.123x_{1}^{2}\\ -0.113x_{2}^{2}-0.090x_{1}x_{3} \end{array}$$

(4) $$\begin{array}{r} -log_{10}\left(100-Y_{2}\right)=-1.50-0.111x_{1}+0.060x_{1}x_{2}\\ +\;0.131x_{1}x_{3} \end{array}$$

Analysis of variance (ANOVA, Tables 7, 8) indicates that second-order polynomial model is adequate to represent the actual relationships between the responses (**LGO** conversion, *Y*_1_, and enzyme residual activity, *Y*_2_) and the significant variables (*p* < 0.05). Satisfactory coefficient of determination (*R*^2^ > 0.5, using *least squares* method) and coefficient of cross-validation (*Q*^2^ > 0.5, using the leave-one-out cross-validation) were obtained, showing the *goodness of fit* and the *goodness of prediction*, respectively. Moreover, *F*-tests performed in ANOVA assessing the significance of the regression model (*p* < 0.05) and the *lack of fit* (*p* > 0.05) showed statistical significance of both models and a similar magnitude of replicate errors (*no lack of fit*).

**Table 7:** **Analysis of variance (ANOVA) for the quadratic polynomial model fitted to LGO conversion, ***Y***_**1**_**.

**Source of variation**	**Degrees of freedom**	**Sum of squares**	**Mean square deviation**	**Standard**	**Significance**
Regression	6	1.498	0.249	0.500	0.000^a^
Residuals	31	0.312	0.010	0.100	
Lack of fit (model error)	11	0.133	0.012	0.110	0.270^b^
Pure error (replicate error)	20	0.179	0.008	0.095	
*R*^2^ / $R_{adj.}^{2}$^c^	0.828/0.794				
*Q*^2^	0.733				

a *Significant at the level 95%*.

b *No lack of fit*.

c *R^2^ adjusted for degree of freedom*.

**Table 8:** **Analysis of variance (ANOVA) for the quadratic polynomial model fitted to enzyme residual activity, ***Y***_**2**_**.

**Source of variation**	**Degrees of Freedom**	**Sum of Squares**	**Mean square Deviation**	**Standard**	**Significance**
Regression	5	0.583	0.117	0.342	0.000^a^
Residuals	31	0.282	0.009	0.095	
Lack of fit (model error)	12	0.144	0.012	0.109	0.162^b^
Pure error (replicate error)	19	0.139	0.007	0.085	
*R*^2^ / $R_{adj.}^{2}$^c^	0.674 / 0.621				
*Q*^2^	0.50				

a *Significant at the level 95%*.

b *No lack of fit*.

c *R^2^ adjusted for degree of freedom*.

#### 3.3.3. Model interpretation

Model interpretation plays an important role in DOE. Model coefficients given by the Equations (**3**) and (**5**) are unscaled in order to enable the direct application of equations without calculating the corresponding code level of each variable. However, when scaled and centered (referring to the coded −1 to +1 unit), the coefficients values are useful for model interpretation (see the values in the Table 5, 6).

**Figure 3:**
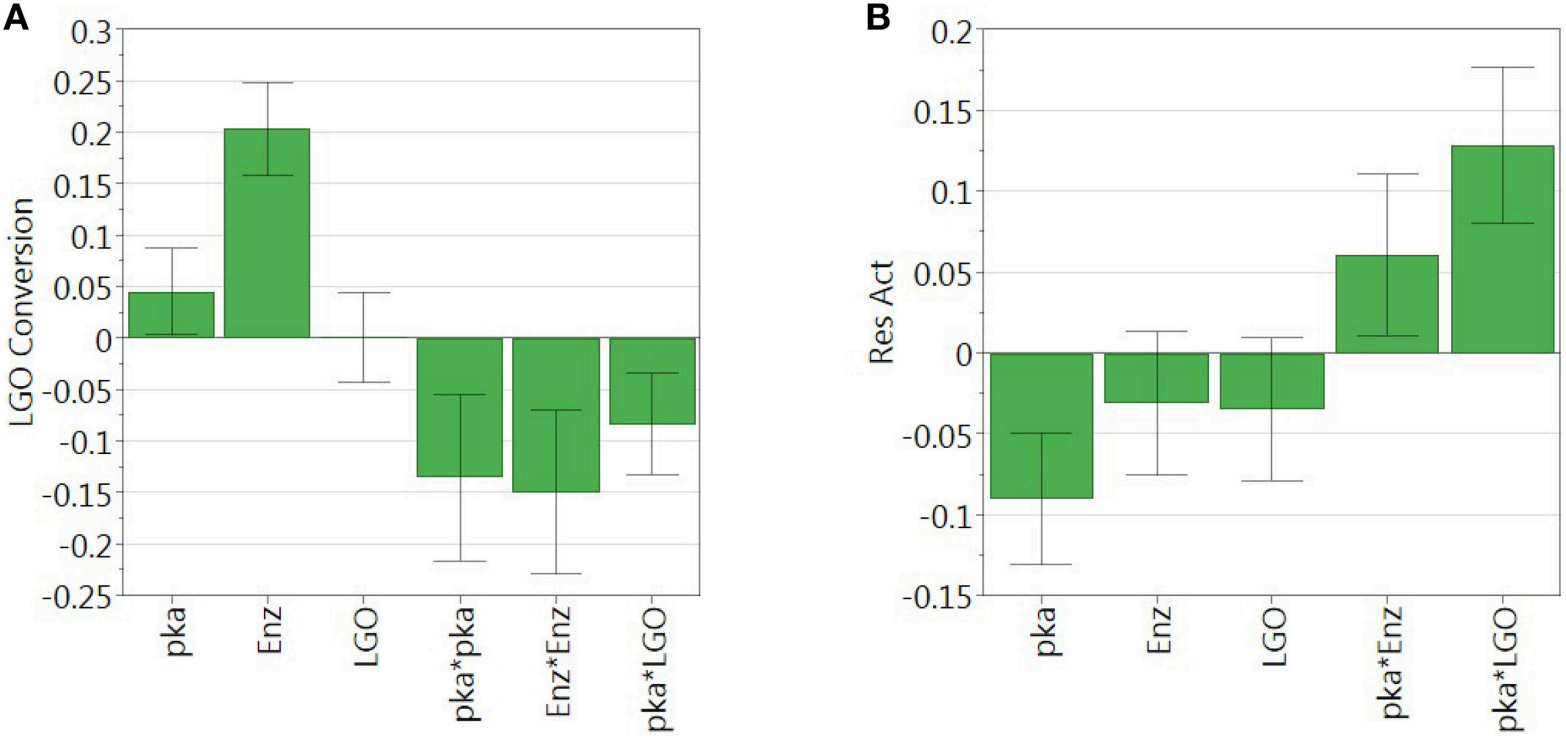
**Regression coefficient of (A) LGO conversion model and (B) enzyme residual activity model**. Model coefficients are scaled and centered.

Figure 3 shows the graphical representation of the coefficient scaled and centered of the models of **LGO** conversion and enzyme residual activity, where positive values indicate a synergistic effect on the response while the negative indicate an antagonistic effect.

As can be observed, enzyme loading (*x*_2_) is the most significant variable affecting positively the conversion of **LGO** (*Y*_1_), being observed as well a negative quadratic effect mainly by the solid buffer pka (*x*_1_) and enzyme loading (*x*_2_). **LGO** concentration (*x*_3_) affects the conversion of **LGO** only due to its interaction with the pka variable (*x*_1_*x*_3_). These results were expected, since increasing the enzyme loading more enzyme units are available to convert the substrate, thus, higher is the conversion of **LGO**. Of course, there are economic restraints in using high enzyme loading which should be taken into account in the optimization. On the other side, the effect of pka (equivalent to the pH in aqueous solutions) on the enzyme activity is widely reported in literature.

The model of the enzyme residual activity (*Y*_2_) is more complex. The solid buffer pka (*x*_1_) proved to have a significant negative effect as well as interacts with the enzyme loading and the **LGO** concentration (*x*_1_*x*_2_ and *x*_1_*x*_3_). The complexity of the enzyme residual activity and existence of interactions is understandable and expected when taking into account all possible mechanisms of activation and inactivation of an enzyme.

**Figure 4:**
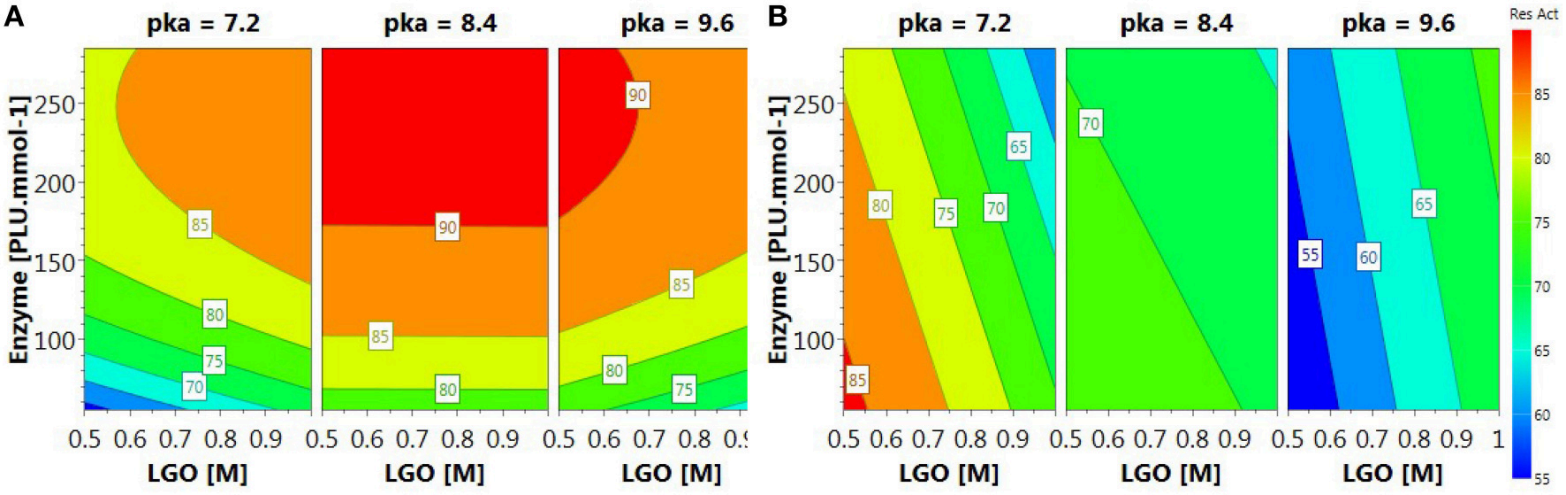
**Contour plots of (A) LGO conversion and (B) enzyme residual activity**.

The relationships between variables and responses can be better understood by examining the contour plots (Figure 4) generated from the models predicting the **LGO** conversion and enzyme residual activity responses (Equations 3 and 5, respectively). The contour plots can provide the contour lines of the independent variables (*x*_*i*_) that have the same response value *Y*_*i*_.

Contour plot of **LGO** conversion (Figure 4A) shows that high values (> 90%) can be reached at higher enzyme loading (175 PLU.mmol^-1^) and pka's (>8.4). However, these conditions corresponds to the lowest enzyme residual activity (Figure 4B). The maximal value for such response (> 85%) was observed at low pka (MOPS, 7.2), low enzyme loading (<100 PLU.mmol^-1^). Therefore, a trade-off between responses should be placed in order to attain the optimal conditions.

#### 3.3.4. Use of regression model for attaining optimal conditions

The optimal conditions were determined using a Nelder-Mead Simplex algorithm. This method computes the variable values (*x*_1_, *x*_2_, and *x*_3_) that minimizes simultaneously the normalized distance to target values of responses and DPMO (Defects Per Million Opportunities outside specifications). DPMO gives information about robustness to small disturbances introduced by the precision specified for the factors. The optimal conditions to obtain at least 80% of **LGO** conversion (*Y*_1_) and enzyme residual activity (*Y*_2_) were found to be: pka = 7.5 (HEPES), **LGO** concentration = 0.50 M and enzyme loading = 194 PLU.mmol^-1^. The advantage of using HEPES as solid buffer, in contrast to MOPS (pka = 7.2), is its easier recovery for reuse in a consecutive batch, since it does not form a gel.

**Figure 5:**
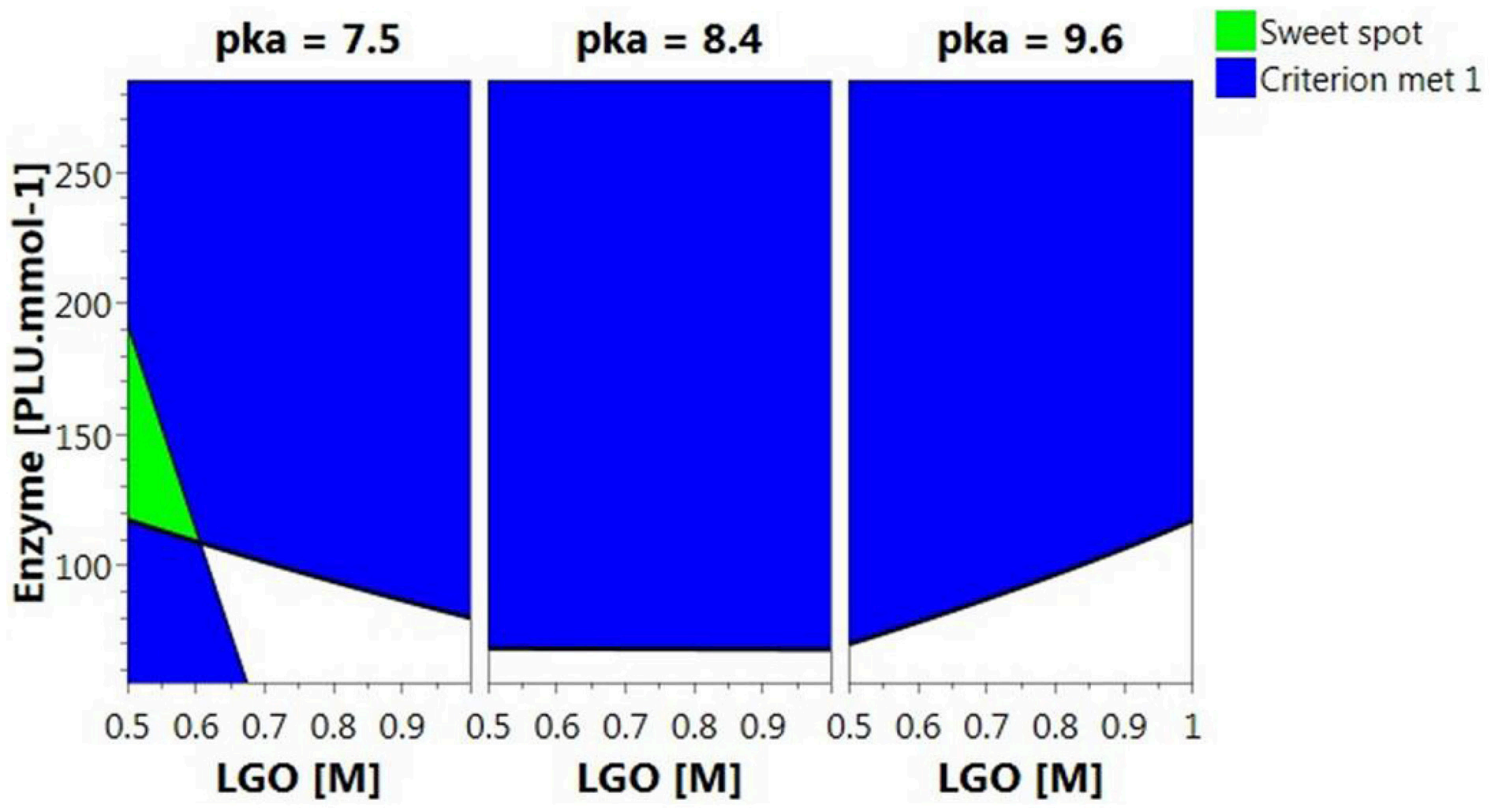
**4D ***Sweet Spot*** plot**. Green color indicates the “sweet spot,” where both responses are at least 80%; blue indicates the area where the criteria fails for one of the responses and white indicates the area where none of the responses are within the selected range.

Figure 5 shows the *Sweet Spot* plot when setting 80% as the minimum value for both responses. As can be observed, the criteria (area highlighted in green) were exclusively met at low pka (HEPES, pka = 7.5) and **LGO** concentration (<0.57 M).

**Figure 6:**
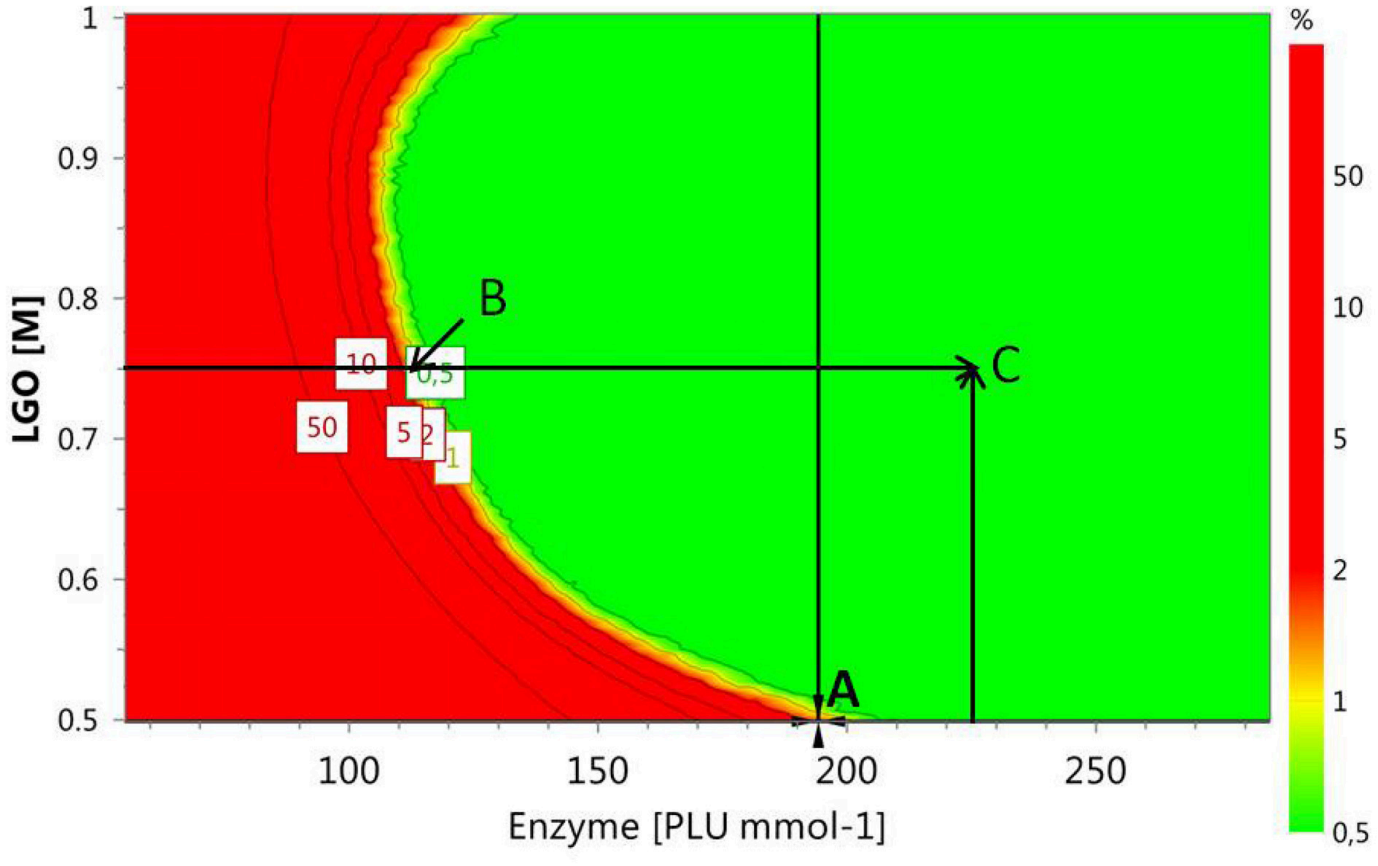
**Design space plot**. Contours indicate the risk of failure (%) in the specifications fulfilling (**LGO** conversion > 80%, pka_*HEPES*_ = 7.5). Green color indicates the area where the risk of failure is lower (< 1%), while the red indicates a higher risk of failure (> 2%). Point *A*- optimum identified by a Nelder-Mead Simplex algorithm (194 PLU.mmol^-1^ and **[LGO]** = 0.50 M), Point *B* - optimum identified in an earlier publication (Flourat et al., 2014) (113 PLU.mmol^-1^ and **[LGO]** = 0.75 M); Point *C* - point with a zero risk level (227 PLU.mmol^-1^ and **[LGO]** = 0.75 M).

Alternative optimal conditions can be explored using a *design space* (*DS*) plot. This plot uses Monte Carlo simulations for risk analysis, estimating the volume in the experimental design region where it can be expected that all specifications are fulfilled at a specific risk level. Figure 6 shows the *DS* plot using as specification a high **LGO** conversion (*Y*_1_ > 80%). However, it should be taken into account that the lower enzyme residual activity, the greater **LGO** conversion (>80%). Three points were identified using this plot. Point A is the optimum identified by a Nelder-Mead Simplex algorithm, Point *B* uses a lower enzyme loading to convert at least 80% of **LGO** (both with a risk level between 1 and 2%) and Point *C* illustrates a condition where no risk is taken to obtain 80% as minimum value for both responses, in spite of using a high enzyme loading.

The advantage of using the conditions expressed by the point *B* is the low enzyme loading (113 PLU.mmol^-1^) necessary to obtain an acceptable **LGO** conversion (80%), which represents an economic saving and increases the viability of the process at industrial scale. The point *B* coincides with the optimal point identified by the authors in an earlier publication (Flourat et al., 2014), however, using the OVAT method.

### 3.4. External model validation

The internal validity of the predicting model was assessed by the Q^2^ coefficient, obtained by leave-one-out cross-validation. Points *A*, *B*, and *C* were used for an additional external validation.

As shown in Table 9, the experimental responses are in agreement to the ones predicted by the model, thus, the model validity can be inferred within the region (ranges of variables) studied in this work.

**Table 9:** **Runs performed for external model validation**.

**Run**	**Factor**			**Estimated response**		**Experimental response**	
	***x*_1_ (pka)**	***x*_2_(PLU.mmol^-1^)**	***x*_3_ (M)**	***Y*_1_**	***Y*_2_**	***Y*_1_**	***Y*_2_**
*A*	HEPES (7.5)	194	0.50	83.6 ± 4.1	80.6 ± 3.4	79.5	80.4
*B*	HEPES (7.5)	113	0.75	82.9 ± 2.5	76.1 ± 2.8	84.0	74.3
*C*	HEPES (7.5)	227	0.75	89.5 ± 4.5	71.6 ± 3.5	94.0	69.4

### 3.5. Enzyme recyclability and HBO production

The possibility of reusing the enzyme under point *B* conditions at multigram scale was shown by the authors in an early publication (Flourat et al., 2014), being obtained high conversion of **LGO** in the first two cycles (> 80% per cycle). Scale up of this process was performed with 10 g of **LGO** per oxidation cycle. Under such conditions, conversions of **LGO** were comparable to those observed at the 500 mg scale (1st cycle: 83 and 81% respectively). The reaction mixture was then combined and subjected to the acid hydrolysis. Finally, after concentration to dryness, the crude mixture was easily purified by column chromatography and provided pure HBO in 67% yield (overall yield for the two 2-h oxidation cycles). ^1^H and ^13^C NMR spectras can be found in our early publication (Flourat et al., 2014).

## 4. Conclusion

RSM has proven to be adequate for the optimization of the enzymatic Baeyer-Villiger oxidation of **LGO**, providing, in addition, a better understanding of the individual and mutual effects of buffer pka, enzyme loading and **LGO** concentration on the overall reaction efficiency (measured as conversion of **LGO**) as well as on the enzyme recyclability (measured as enzyme residual activity). Enzyme loading and solid buffer pka were found to be important variables to the **LGO** conversion while for the enzyme residual activity, only the pka and all their interactions were significant. **LGO** concentration influences both responses by their interaction with the enzyme loading and solid buffer pka.

An antagonist effect of the variables on both responses was observed, thus, being necessary to establish a compromise to attain the optimal conditions. Such conditions were found to be: solid buffer pka = 7.5, **[LGO]** = 0.75 M and 113 PLU.mmol^-1^ for the lipase. Under these conditions, a high conversion (> 80%) was obtained in two consecutive batch at multigram scale.

The statistical models obtained by RSM for each response, represented by Equations (**3**) and (**5**), enable the prediction of **LGO** conversion and enzyme residual activity, respectively, at different conditions of pka, enzyme loading and **LGO** concentration. The validity of the model was confirmed (*p* < 0.05), being observed a good agreement between experimental and predicted values.

## Author contributions

FA conceived and supervised the research project. AT performed the experimental design and its statistical analysis. AF and AP carried-out all the enzymatic reaction and quantified LGO by HPLC. AT done all the enzymatic residual activity tests, while FB quantified by GC-MS. AT drafted the manuscript, all authors were involved in revising it; FA supervised its preparation. All authors have approved and are accountable for the final version of the manuscript.

## Funding

The authors are grateful to Région Champagne-Ardenne, Conseil Général de la Marne and Reims Métropole for financial support.

### Conflict of interest statement

The authors declare that the research was conducted in the absence of any commercial or financial relationships that could be construed as a potential conflict of interest.
