# Supplementary material for: Lipase-Catalyzed Baeyer-Villiger Oxidation of Cellulose-Derived Levoglucosenone into (S)-γ-Hydroxymethyl-α,β-Butenolide: Optimization by Response Surface Methodology
Source: Front Chem. 2016 Apr 19;4:16. doi: 10.3389/fchem.2016.00016 (PMC4835721; doi:10.3389/fchem.2016.00016)
Supplement: Supplementary file 1 [file Presentation1.pdf]

Supplementary Material: Lipase-catalyzed Baeyer-Villiger Oxidation of cellulose-derived Levoglucosenone into (*S*)-γ-hydroxymethyl-α,β-butenolide: Optimization by Response Surface Methodology

A.R.S. Teixeira*, A.L. Flourat, A.A.M. Peru, F. Brunissen, F. Allais
*Correspondence:
A.R.S. Teixeira
andreia.teixeira@agroparistech.fr

1 SUPPLEMENTARY DATA

1.1 Log-Transformation of responses and data normality assessment

In regression analysis, it is advantageous if the data of a response variable are normally distributed, or nearly so. Normal is used to describe a symmetrical, bell-shaped curve, which has the greatest frequency of scores in the middle, with smaller frequencies towards the extremes. The response histogram and the Box-Whisker plot are useful for studying the distributional shape of a response variable and determining the need of a response transformation. Figures S1 and S2 show the response histogram and Box-Whisker plot for **LGO** conversion and Enzyme residual activity, respectively, before and after negative log-transformation.

Histograms of negatively skewed responses are observed before transformation (Figures S1a and Fig. S2e, being obtained a nearly normal distribution after a negative log-transformation (Figures S1b and Fig. S2f). With this transformation each measured value is subtracted from the maximum value (100, for variables expressed in percentages), and then the negative logarithm is formed. Thus, a negative log-transformation of a response Y_i can be expressed as:

$$-log_{10}(100 - Y_i) \quad \text{(Eqn S1)}$$

Figs. S1c, S1d, S2g, S2h display Box-Whisker plots corresponding to the respective response histograms. The Box-Whisker plot comprises a rectangular body, the box, and two attached antennae, the whiskers. In the box, the lowest horizontal line depicts the lower quartile ($Q_{25\%}$) and the upper line the upper quartile ($Q_{75\%}$). The lower and upper whisker denote the 5 and 95 percentiles of the distribution, indicating the variability outside the upper and lower quartiles. Whenever the two whiskers are of similar length, the distribution of the data is roughly normal.

In addition, the normality of the data after response transformation can be checked by plotting the normal probability plot of the residuals. The normal probability plot is a graphical technique for assessing whether

or not a data set is approximately normally distributed. Fig. S3 displays the normal probability plot for the empirical models for *conversion of **LGO*** and *enzyme residual activity*. The straight line in the plot of the residuals represents a normal distribution, supporting the adequacy of the least-squares fit.

2 MODEL FITTING

When fitting a regression model the most important diagnostic tool consists of the two parameters R^2 (coefficient of determination) and Q^2 (cross-validation).

R^2 measures the *goodness of fit*, indicating how well the regression model can be made to fit the raw data. When R^2 is 1, a perfect model is obtained where all points are situated on a diagonal of a graph representing the observed values vs predicted. Fig. S4 represents the Observed vs Predicted Plot for both responses: ***LGO** conversion* and *enzyme residual activity*.

A much better indication of the usefulness of a regression model is given by the Q^2. This parameter measures the *goodness of prediction*, and estimates the predictive power of the model. Like R^2, Q^2 has as upper bound 1. Both should be high ($>$ 0.5), and preferably not separated by more 0.2 - 0.3. A substantially larger difference constitutes a warning of an inappropriate model. A third parameter is called *model validity*. It is based on the *lack of fit* test carried out as part of the analysis of variance (ANOVA) evaluation. The higher the numerical value the more valid the model is, and a value above 0.25 suggests a valid model. Finally, a last diagnostic tool called *reproducibility*. This performance indicator is a numerical summary of the variability in replicates. The higher the numerical value the smaller the replicate error is in relation to the variability seen across the entire design. A small value of *reproducibility* ($<$ 0.5) indicates a large experimental error (aka *pure error*) and poor control of the experimental procedure.

Fig. S5 represents the summary of fit plot of the ***LGO** conversion* and *enzyme residual activity* models. As observed, both models are valid and well fitted and predicted by the second-order polynomial equation (eqn. 1), presenting, in addition, a good reproducibility.

The fitted models expressed in uncoded variables were represented by the following equations:

$$-log_{10}(100 - Y_1) = -10.3 + 1.83\,x_1 + 5.61 \times 10^{-3}\,x_2 - 0.094\,{x_1}^2 - 1.13 \times 10^{-5}\,{x_2}^2 - 0.278\,x_1x_3 \qquad \text{(Eqn S2)}$$

$$-log_{10}(100 - Y_2) = 2.62 - 0.471\,x_1 + 4.40 \times 10^{-4}\,x_1x_2 + 0.428\,x_1x_3 \qquad \text{(Eqn S3)}$$

The graphical representation of such equations are showed in Figure S6.

3 SUPPLEMENTARY FIGURES

3.1 Figures

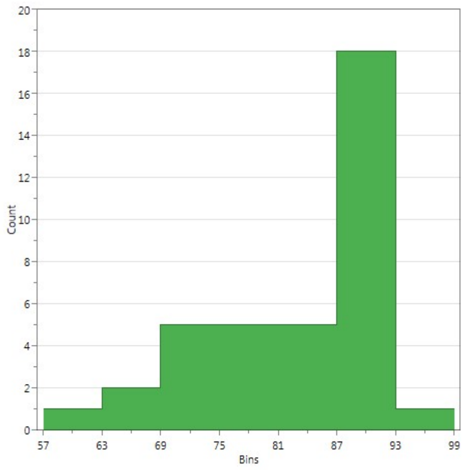

(1a)

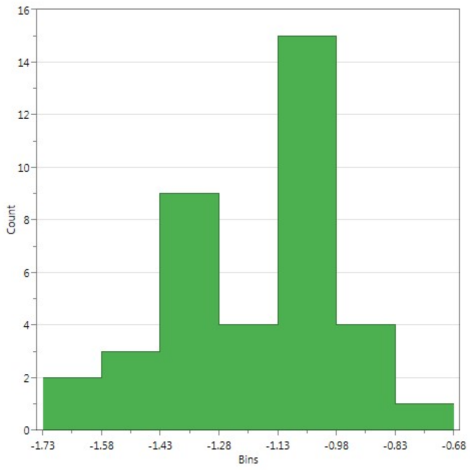

(1b)

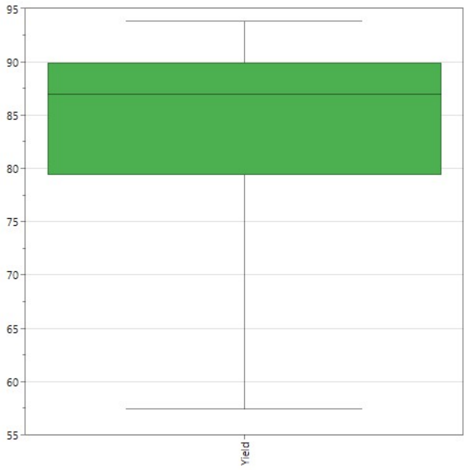

(1c)

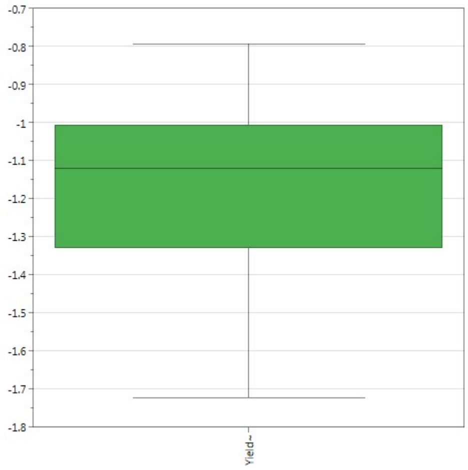

(1d)

Figure S1. Histogram and Box Whisker of ***LGO*** *conversion* before (a),(c) and after (b),(d) negative log-transformation

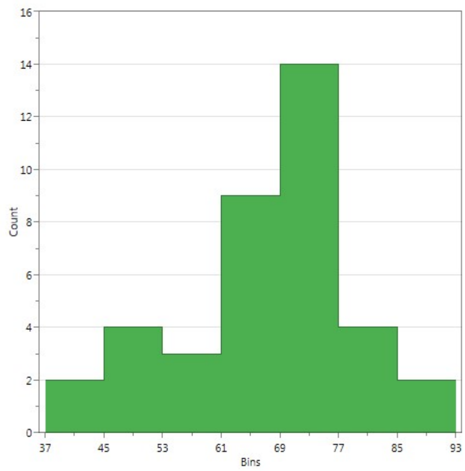

(2e)

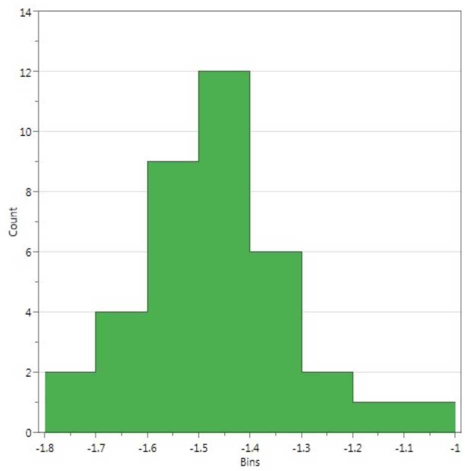

(2f)

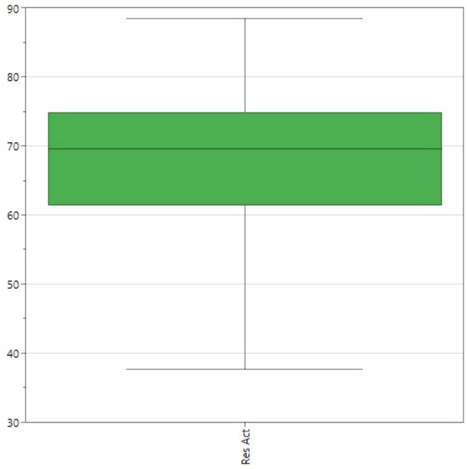

(2g)

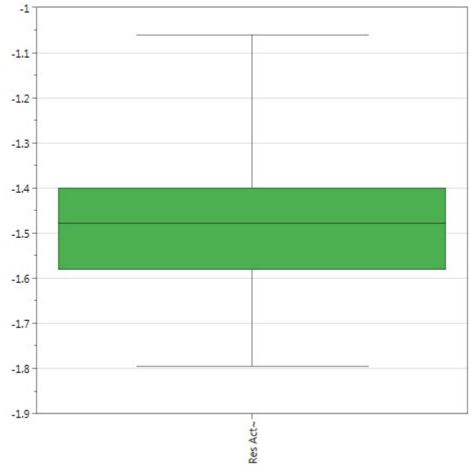

(2h)

Figure S2. Histogram and Box Whisker of *enzyme residual activity* response (e), (g) before and (f), (h) after negative log-transformation

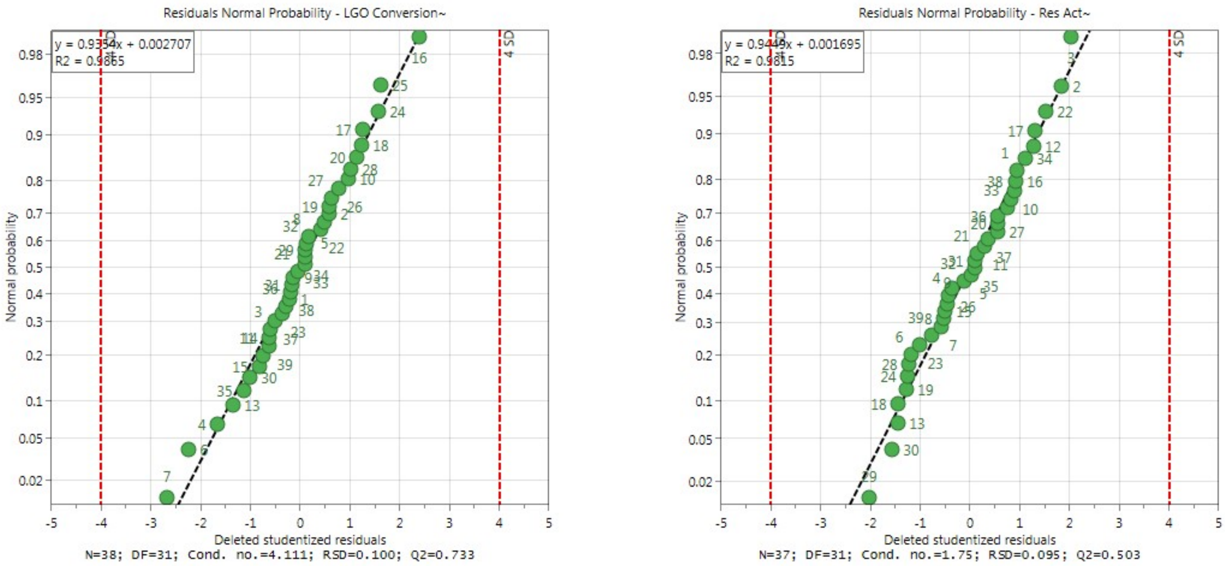

(3i) **(3j)**

Figure S3. Normal probability plot of the residuals for the responses: (i) ***LGO*** *conversion* and (j) *enzyme residual activity*

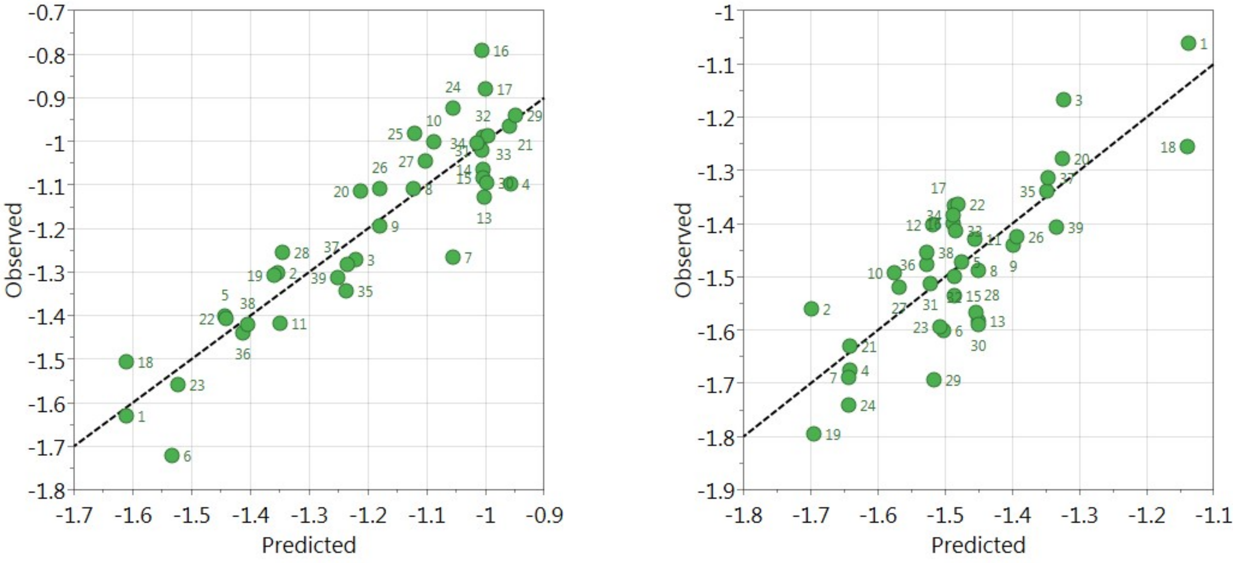

(4k) **(4l)**

Figure S4. Observed vs Predicted Plot for (k) ***LGO*** *conversion* and (l) *enzyme residual activity*. Experimental data points are identified by the run number

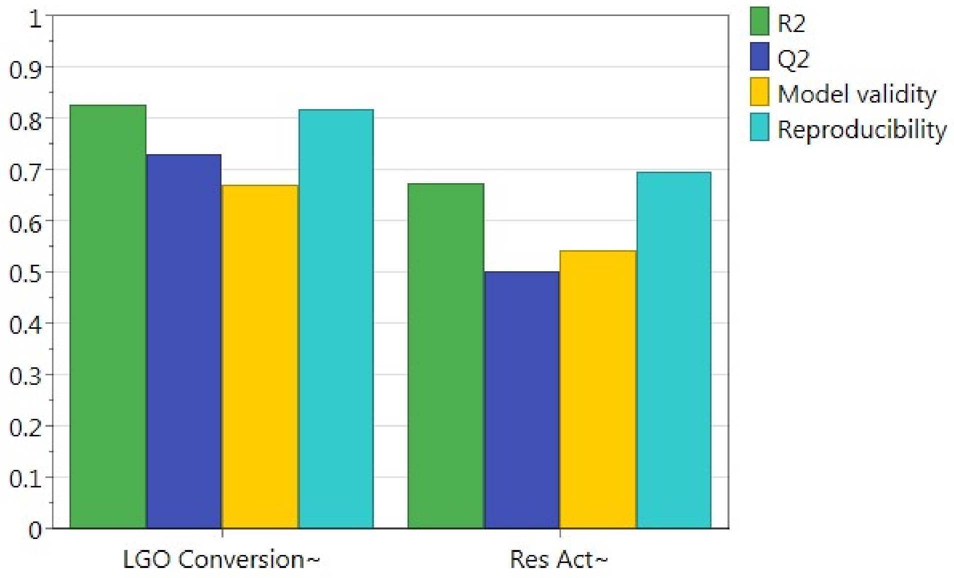

Figure S5. Summary of fit plot of the *LGO conversion* and *enzyme residual activity* (Res Act) models

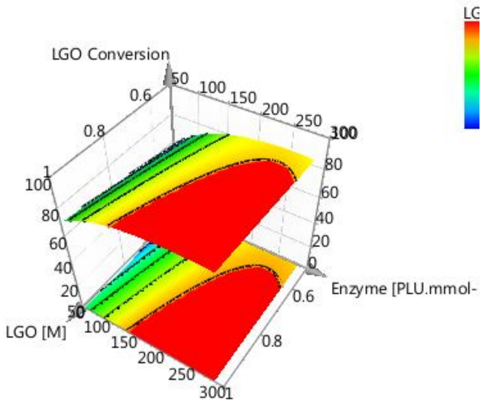

(6m)

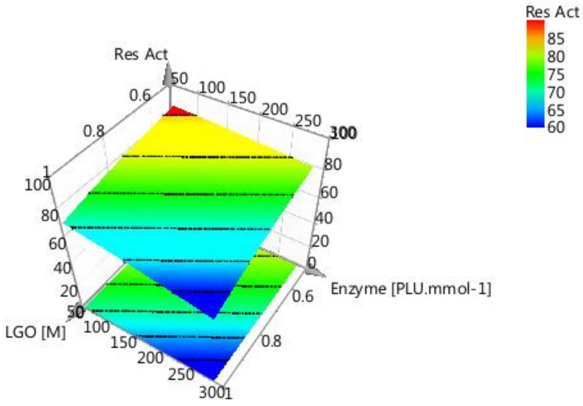

(6n)

Figure S6. Surface plots of (m) **LGO** conversion and (n) enzyme residual activity, fixing the pka at 7.2 (MOPS)
